# Novel Functional Analysis for Pathogenic Proteins of *Bursaphelenchus xylophilus* in Pine Seed Embryos Using a Virus Vector

**DOI:** 10.3389/fpls.2022.872076

**Published:** 2022-04-25

**Authors:** Haru Kirino, Ken-ichi Konagaya, Ryoji Shinya

**Affiliations:** ^1^School of Agriculture, Meiji University, Kawasaki, Japan; ^2^Forest Bio-Research Center, Forestry and Forest Products Research Institute, Hitachi, Japan

**Keywords:** pine wilt disease, pine wood nematode, exogenous gene expression, hypersensitive responses, pathogenesis-related genes, *apple latent spherical virus*

## Abstract

Pine wilt disease (PWD), which is caused by the pine wood nematode *Bursaphelenchus xylophilus*, is among the most serious tree diseases worldwide. PWD is thought to be initiated by sequential excessive hypersensitive responses to *B. xylophilus*. Previous studies have reported candidate pathogenic molecules inducing hypersensitive responses in pine trees susceptible to *B. xylophilus*. The functions of some of these molecules have been analyzed in model plants using transient overexpression; however, whether they can induce hypersensitive responses in natural host pines remains unclear due to the lack of a suitable functional analysis method. In this study, we established a novel functional analysis method for susceptible black pine (*Pinus thunbergii*) seed embryos using transient overexpression by the *Apple latent spherical virus* vector and investigated five secreted proteins of *B. xylophilus* causing cell death in tobacco to determine whether they induce hypersensitive responses in pine. We found that three of five molecules induced significantly higher expression in pathogenesis-related genes ( *p* < 0.05), indicating hypersensitive response in pine seed embryos compared with mock and green fluorescence protein controls. This result suggests that tobacco-based screening may detect false positives. This study is the first to analyze the function of pathogenic candidate molecules of *B. xylophilus* in natural host pines using exogenous gene expression, which is anticipated to be a powerful tool for investigating the PWD mechanism.

## Introduction

Pine wilt disease (PWD), which is caused by the pine wood nematode *Bursaphelenchus xylophilus*, is a serious threat to pine forests worldwide (Kiyohara and Tokushige, 1971; Yi et al., 1989; Burgermeister et al., 1999; Abelleira et al., 2011). The mechanism by which *B. xylophilus* kills host pine trees has been extensively studied during the past 50 years. Based on physiological and histological observation, sequential excessive hypersensitive responses to the spread of *B. xylophilus* through the tree are thought to lead, eventually, to the death of susceptible pine trees (Myers, 1988; Futai, 2013). The expression levels of pathogenesis-related (PR) genes, that is the landmark of hypersensitive responses, have been found to be much higher in susceptible trees inoculated with nematodes than in resistant trees (Hirao et al., 2012). Furthermore, Yamaguchi et al. (2019) showed that all of pine seedling, that increased the number of *B. xylophilus* and expression levels of PR genes after nematodes inoculation, exhibited external symptoms. Accordingly, identifying *B. xylophilus* molecules that induce hypersensitive responses in host pine trees is important for predicting PWD occurrence, and the range of candidate molecules has been narrowed down using genomic, transcriptome, and proteome methods (Kikuchi et al., 2011; Shinya et al., 2013, 2021; Palomares-Rius et al., 2015; Cardoso et al., 2016, 2021; Ding et al., 2016; Espada et al., 2016, 2018; Filipiak et al., 2020; Silva et al., 2021). Recent functional analyses of these candidate molecules have mainly applied RNAi in *B. xylophilus* or examined the expression of its virulence factors in plants (Ma et al., 2011; Huang et al., 2019; Qiu et al., 2019). Because *B. xylophilus* RNAi effects are generally transient and weak, functional analysis using plants appears to be a more suitable approach for this research (Park et al., 2008).

The overexpression of foreign genes in plants is a major biotechnological tool in molecular functional analysis. For example, leaf-disk assays based on transient overexpression in the model plant *Nicotiana benthamiana* have been applied to investigate whether pathogenic candidate molecules of *B. xylophilus* induce cell death (Hu et al., 2019; Kirino et al., 2020; Shinya et al., 2021). Such studies have successfully narrowed the field of candidate molecules; however, whether they induce hypersensitive responses in natural pines remains unclear. Therefore, the next challenge is to develop a method for expressing *B. xylophilus* protein in pine trees and to perform functional analysis of the pathogenic candidate molecules. Because transgenic pine seedling production requires substantial time and effort, they are unsuitable for screening *B. xylophilus* pathogenic candidates. Therefore, in this study, we focused on pine seed embryos instead of pine seedlings. Exogenous proteins such as green fluorescent protein (GFP) can be transiently expressed in pine seed embryos using the *apple latent spherical virus* (ALSV) vector (Konagaya, 2017). ALSV is a small spherical virus (diameter, 25 nm) originally isolated from apple trees (Li et al., 2000, 2004) and has two ssRNA species (pEALSR1 and pEALSR2). ALSV-based vectors can be used to infect experimentally a broad range of plant species without causing symptoms, effectively inducing stable virus-induced expression of foreign genes in plants (Takahashi and Yoshikawa, 2008). Therefore, we hypothesized that an ALSV-based method is suitable for identifying pathogenic molecules in *B. xylophilus*.

To evaluate the effectiveness of this method using pine seed embryos with ALSV for the functional analysis of pathogenic molecules in *B. xylophilus*, we selected five proteins secreted by *B. xylophilus* causing cell death in tobacco (Kirino et al., 2020; Shinya et al., 2021) and expressed them in susceptible black pine (*Pinus thunbergii*) seed embryos through transient overexpression using the ALSV vector. Because *B. xylophilus* protein expression induced using the ALSV vector is transient and insufficient for inducing cell death, genetic markers are more suitable for evaluating host responses. Then, we examined whether hypersensitive response could be induced through the expression of each *B. xylophilus* protein based on expression of PR-1b (enigmatic biochemical function), PR-2 (beta-1,3-glucanase), PR-3 (classes I and IV chitinase), PR-4 (chitinase types I and II), PR-5 (thaumatin-like protein), and PR-6 (type II proteinase inhibitor family protein) in pine seed embryos because the expression of these genes was induced more quickly and to a higher level in susceptible than in resistant trees following infection by a virulent isolate of *B. xylophilus*, suggesting that the expression of these PR genes is good indicators of hypersensitive response that precede pine wilt (Hirao et al., 2012). To our knowledge, this is the first report of functional analysis using recombinant pathogenic candidate molecules of *B. xylophilus* in host pines. This method is anticipated to be a powerful tool for investigating the PWD mechanism.

## Materials and Methods

### Nematode Strains and Culturing

We used the virulent Ka4 isolate of *B. xylophilus*, which was propagated on the BC-3 strain of the fungus *Botrytis cinerea*, grown at 25°C on malt extract agar (MEA) plates (Difco Laboratories) containing 100 μg/ml chloramphenicol in 90-mm petri dishes.

### Nematode Sterilization

Nematodes grown for 3–5 days in mixed culture were collected into 15-mL tubes containing sterile water. The tubes were centrifuged at 2,000 rpm for 2 min. The supernatant was discarded, with nematodes retained at the bottom of the tube. We added 5 ml of a solution consisting of 0.002% mercuric chloride, 0.0025% sodium azide, and 0.001% Triton X-100 in sterile water; the solution was allowed to stand for 10 min. The nematodes were washed with sterile water three times. Then, a nematode solution in 5 ml sterile water containing 5 μl of 1,000 ppm streptomycin was incubated at 12°C overnight. The nematodes were again washed with sterile water three times and then re-suspended in sterile water and used for inoculation into pine seed embryos.

### Pine Seed Sterilization

We used a PWD-susceptible black pine Taga-1 isolate of *P. thunbergii* in this study. Pine seeds were immersed in 70% ethanol and shaken for 3 min. Floating seeds were discarded. The remaining seeds were immersed in 5% hydrogen peroxide solution in sterile water containing a drop of Tween 20 and shaken for 10 min. The seeds were washed with sterile water five times, immersed in sterile water at 4°C for 1 week, and then used for nematode or biolistic inoculation.

### Nematode Inoculation to Pine Seed Embryos

For each sterilized pine seed, the seed coat and endosperm were removed and the embryo was extracted using tweezers and knife. These pine seed embryos were sown on a 60-mm mGD plate (Konagaya et al., 2020) containing 10 μg/ml benomyl and 100 μg/ml chloramphenicol. Twelve pine seed embryos were inoculated approximately 500 nematodes per plant under aseptic conditions, and six were not inoculated nematodes (mock control) with one replicate per experiment. The embryos were placed into a growth chamber (25°C, 24 h light).

### Nematode Total RNA Extraction and cDNA Synthesis

Mixed-stage nematodes grown for 5 days on MEA plates were collected with sterile water, sedimented by centrifugation at 2,500 rpm for 2 min, and re-suspended in fresh sterile water. This procedure was repeated three times. After the final wash, the nematode pellet was homogenized using a mortar and pestle after freezing with liquid nitrogen. Total RNA was extracted from homogenized nematodes and purified using the RNeasy Mini Kit (Qiagen), according to the manufacturer’s protocol. Reverse transcription was performed using 1 μg of total RNA and the PrimeScript II first-strand cDNA Synthesis Kit (Takara Bio Inc.), according to the manufacturer’s protocol. The transcribed cDNA was adjusted to a concentration of 200 ng/μL and subjected to PCR amplification.

### Construction of ALSV Vectors

The candidate proteins (Table 1) were proteins secreted by *B. xylophilus* that were found to cause tobacco cell death in our previous studies (Kirino et al., 2020; Shinya et al., 2021). The sequence of *Bx-CPI* included signal peptide because it induced cell death in *N. benthamiana* only when signal peptide was present. Signal peptides of the other proteins were removed. All sequences including the GFP control were amplified by PCR from cDNA extracted from *B. xylophilus* or pRSET-EmGFP (Thermo Fisher Scientific). All PCR assays were performed using Takara PrimeSTAR GXL DNA polymerase, according to the manufacturer’s protocol (Takara Bio Inc.). The PCR components were as follows: 10 μl 5 × Prime STAR GXL buffer, 4 μl dNTP Mixture (2.5 mM each), 1 μl forward primer (10 μM), 1 μl reverse primer (10 μM), 1 μl Prime STAR GXL Polymerase, 1 μl cDNA template (200 ng/μL), and 32 μl H_2_O. The cycling conditions were as follows: 35 cycles at 98°C for 10 s, 55°C for 15 s, and 68°C for 60 s/kb. The primers were designed using the Primer 3 Plus software (http://www.bioinformatics.nl/cgi-bin/primer3plus/primer3plus.cgi). The primers are listed in Table 2. The DNA products from *B. xylophilus* were inserted into the pEALSR2L5R5 vector (Yamagishi and Yoshikawa, 2013) using the In-Fusion HD Cloning Kit (Takara Bio Inc.) under the following conditions: 37°C for 15 min and 42°C for 15 min. The amplified GFP was digested with *Xho* I and *Bam* HI, and ligated to pEALSR2L5R5 vectors restricted with the same enzymes following previous research (Li et al., 2004). The resulting clones were designated pEALSR2-BxTH1, pEALSR2-BxTH2, pEALSR2-BxCPI, pEALSR2-BxGH30, and pEALSR2-BxGST. Gene sequences were confirmed by sanger sequence analysis using primer pair; Virus FP and Virus RP in Table 2 (Yamagishi et al., 2014). Both pEALSR1 and the constructed pEALSR2 that ALSV consists of were transformed in *Escherichia coli* JM109 and purified from large-scale cultures using the Plasmid Mini Kit (Qiagen); each was mixed at a concentration of 0.5 μg/μL and then mechanically inoculated into *Chenopodium quinoa* leaves at the eight true leaf stage for propagating ALSV vectors (25°C, 16 h:8 h light:dark). Three independent plants were inoculated per vectors. Leaves with chlorotic spots were homogenized by shaking twice at 1,500 rpm for 3 min with 15 iron balls (diameter, 5 mm) in 15-mL tubes, and RNA was extracted using the RNeasy Mini Kit.

**Table 1:** Candidate pathogenic molecules secreted by *Bursaphelenchus xylophilus.*

Gene DB accession	Full-length base sequences (bp)	Protein annotation
BUX.s00036.89	441	thaumatin-like (Bx-TH1)
BUX.s00036.92	258	thaumatin-like (Bx-TH2)
BUX.s00351.347	375	cysteine protease inhibitor-like (Bx-CPI)
BUX.s00647.112	717	glutathione s-transferase (Bx-GST)
BUX.s00713.1066	1,545	o-glycosyl hydrolase family 30 (Bx-GH30)

**Table 2:** List of primers used in this study.

Primer name	Sequence (5′ to 3′)	References
Bx-TH1 FP	TTCACACTCGAGCCCATGGGATATACGAGCTTCG	−
Bx-TH1 RP	TAGCAGGGATCCCCCAGGACAGTAGATGACGTAG	−
Bx-TH2 FP	TGATTTCACACTCGAGATGGAGTTCTCCTTCGAGA	−
Bx-TH2 RP	CTTCTAGCAGGGATCCGCAGAAGTACAAGTCGAAC	−
Bx-CPI FP	TTCACACTCGAGCCCATGTTGTTCAAAGTTACTGTG	−
Bx-CPI RP	TAGCAGGGATCCCCCCTTTTGCTTGACGATTTCGG	−
Bx-GST FP	TGATTTCACACTCGAGATGTTAGAGCTGTATTATTTCA	−
Bx-GST RP	CTTCTAGCAGGGATCCTTGAGTGGCATTGAAATAATTG	−
Bx-GH30 FP	TGATTTCACACTCGAGATGACGCCTTGCAAGAAGG	−
Bx-GH30 RP	CTTCTAGCAGGGATCCACTATTGTTGAAGATGATGGT	−
GFP FP	CCCTCGAGATGGTGAGCAAGGGCGAGGA	Li et al., 2004
GFP RP	CGGGATCCCTTGTACAGCTCGTCCA	Li et al., 2004
Elongation factor-1 alpha FP	GGGAAGCCACCCAAAGTTTT	Hirao et al., 2012
Elongation factor-1 alpha RP	TACATGGGAAGACGCCGAAT	Hirao et al., 2012
PR-2 family FP	CGACAACATTCGCCCCTTCT	Hirao et al., 2012
PR-2 family RP	CTGCAGCGCGGTTTGAATAT	Hirao et al., 2012
PR-4 family FP	CCCCGTTACTGTCAATTGCAT	Hirao et al., 2012
PR-4 family RP	AAAGCGTGACGGTGCGTATT	Hirao et al., 2012
PR-5 family FP	GAACCAGTGCCCATACACAGTCT	Hirao et al., 2012
PR-5 family RP	CCTGCGGCAACGTTAAAAGTC	Hirao et al., 2012
PR-6 family FP	TGCTGGCGGCATCTATTTTA	Hirao et al., 2012
PR-6 family RP	TAACACCTGCGCAAATGCA	Hirao et al., 2012
Virus FP (4F6150(+))	CGATGAATCTCCCTGATAGA	Yamagishi et al., 2014
Virus RP (4R6279(−))	AGAGTAGTGGTCTCCAGCAA	Yamagishi et al., 2014

The underlined sequences indicate restriction sites.

### Biolistic Inoculation of Pine Seed Embryos With ALSV

Biolistic inoculation of pine seed embryos with ALSV-RNAs was performed as described previously (Yamagishi et al., 2010), with modifications. We added 2 mg of gold particles (diameter, 0.6 μm) and 12.5 μl RNase-free H_2_O into a 2-mL microcentrifuge tube. The tube was then vortexed vigorously using a MicroMixer E-36 (Taitec) for 5 min and then sonicated for at least 5 min. During vortexing, 33 μl of 1.5 μg/μL total RNA solution extracted from ALSV-infected *C. quinoa* leaves, 11 μl of 10 M ammonium acetate, and 113 μl of 2-propanol were added to the tube sequentially. After continual vortexing for 7–10 min, gold particles coated with the RNAs were stored at −20°C for 1 h to overnight. The pellet of gold particles was washed gently three times with 1 ml of 100% ethanol and the pellet was re-suspended in 66 μl of 100% ethanol. Pine seed embryos extracted from sterilized seeds were placed on a 90-mm mGD plate containing 10 μg/ml benomyl and 0.4 M maltose and inoculated biolistically with ALSV on the following day. Fifteen pine seed embryos were bombarded with gold particles coated with total RNAs from ALSV-infected leaves per different ALSV vectors, and nine were bombarded with non-coated gold particles (mock control) at a pressure of approximately 1,100 psi using the PDS-1000/He Particle Delivery system (Bio-Rad) with one replicate per experiment. Pine seed embryos were bombarded in two shots, each containing 0.3 mg of particles. Next, the pine seed embryos were placed in a growth chamber (25°C, 24 h light). The next day, pine seed embryos were placed on a 90-mm mGD plate containing 10 μg/ml benomyl and returned to the growth chamber. GFP fluorescence was observed using a MS FLIII fluorescence stereomicroscope (Leica Microsystems) with a GFP2 Plus filter system (excitation filter, 480/40 nm; emission filter, 510 nm). The GFP signal was imaged using a DC300 F digital camera system (Leica Microsystems).

### Quantitative Reverse-Transcription Polymerase Chain Reaction and Normalization

**Figure 1:**
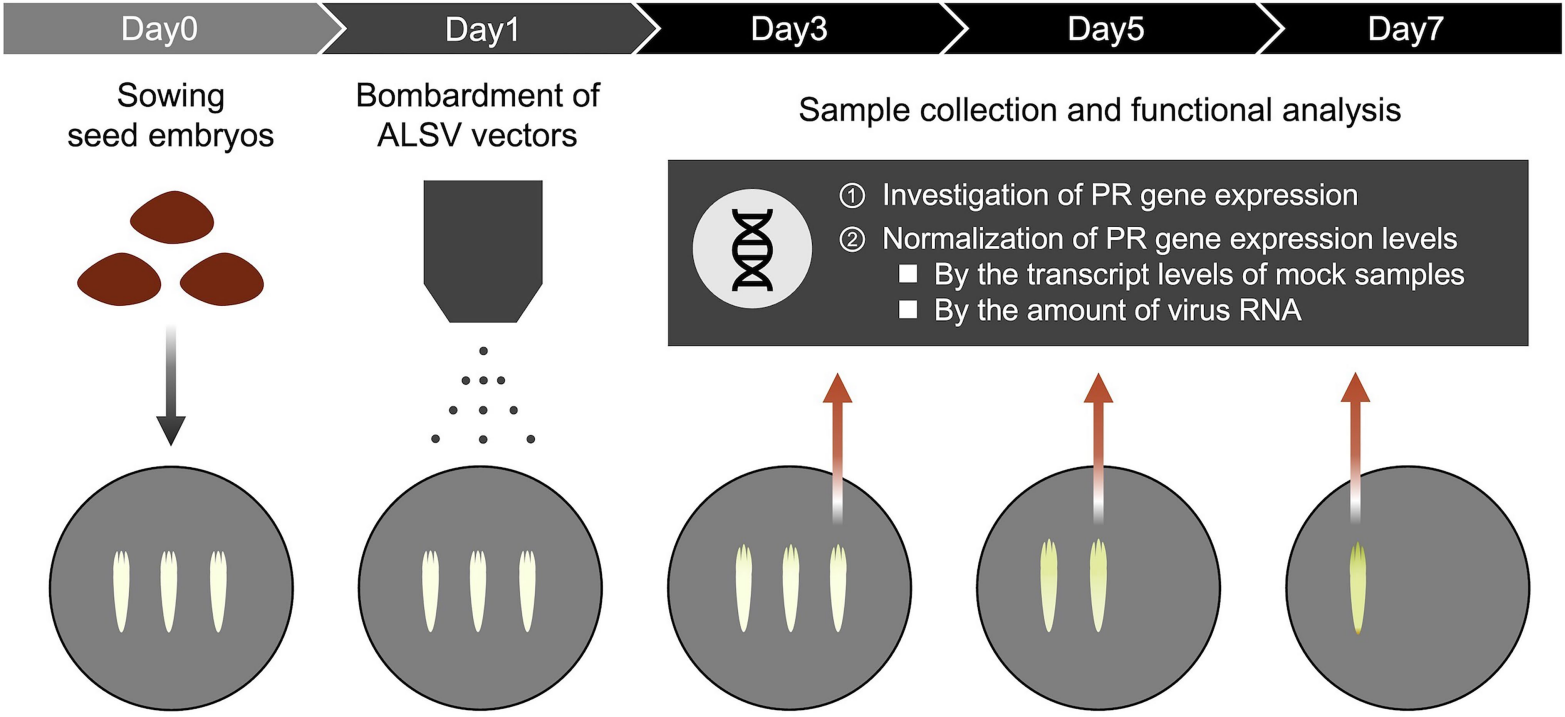
Functional analysis for pathogenic proteins of *Bursaphelenchus xylophilus* in pine seed embryos using a virus vector. Pine seed embryos extracted from sterilized seeds were placed on mGD plate and bombarded with gold particles coated with ALSV or non-coated gold particles (mock control) on the following day. The expression of PR genes was investigated at 3, 5, and 7 dpi after particle bombardment of ALSV vectors using quantitative real-time polymerase chain reaction (qRT-PCR). All PR gene expression levels in pine seed embryos expressing foreign genes were normalized by transcript levels of mock samples and detecting the amount of viral RNA.

For quantitative reverse-transcription polymerase chain reaction (qRT-PCR) analysis, samples were collected from pine seed embryos at 3 and 5 days post-*B. xylophilus* infection (dpi), or at 3, 5, and 7 dpi after particle bombardment with ALSV vectors. Total RNA was isolated from pine seed embryos using the RNeasy Plant Mini Kit (Qiagen) following the manufacturer’s protocol. RT-PCR was performed using total pine seed embryo RNA. Total RNA was reverse-transcribed using the PrimeScript II first-strand cDNA synthesis kit (TaKaRa) according to the manufacturer’s protocol and qRT-PCR was performed using the Power SYBR Green PCR Master Mix (Applied Biosystems) on the StepOnePlus Real-Time PCR System (Applied Biosystems). The PCR mixtures were prepared according to the manufacturer’s instructions and contained 300 nM each of the forward and reverse gene-specific primers and 4 μl of the 20-fold diluted reverse-transcription reaction (total, 5 ng) for a final volume of 20 μl. All reactions were heated to 95°C for 10 min; this denaturation step was followed by 40 cycles of 95°C for 15 s and 60°C for 1 min. The PCR products were subjected to melting curve analysis under the following conditions: 60–95°C at a temperature increment of 1.6°C/s. Elongation factor 1-alpha (FY842441.1) was used as the reference gene for normalizing the transcript profiles. Primer pairs were designed as described previously (Hirao et al., 2012; Table 2). Real-time PCR data were calibrated against the transcript levels of mock samples; the 2^−ΔΔCt^ method was used to quantify relative transcript abundance. Pine seed embryos expressing GFP were used as a control sample to distinguish specific and non-specific induced expression changes. All PR gene expression levels in pine seed embryos expressing foreign genes were normalized by detecting the amount of viral RNA. Primer pairs for detecting the amount of viral RNA were designed as described previously (Yamagishi et al., 2014; Table 2). Figure 1 shows an outline of the experiment method.

## Results

### *PR-2*, *PR-4*, *PR-5*, and *PR-6* Reacted to *B. xylophilus* in Pine Seed Embryos

**Figure 2:**
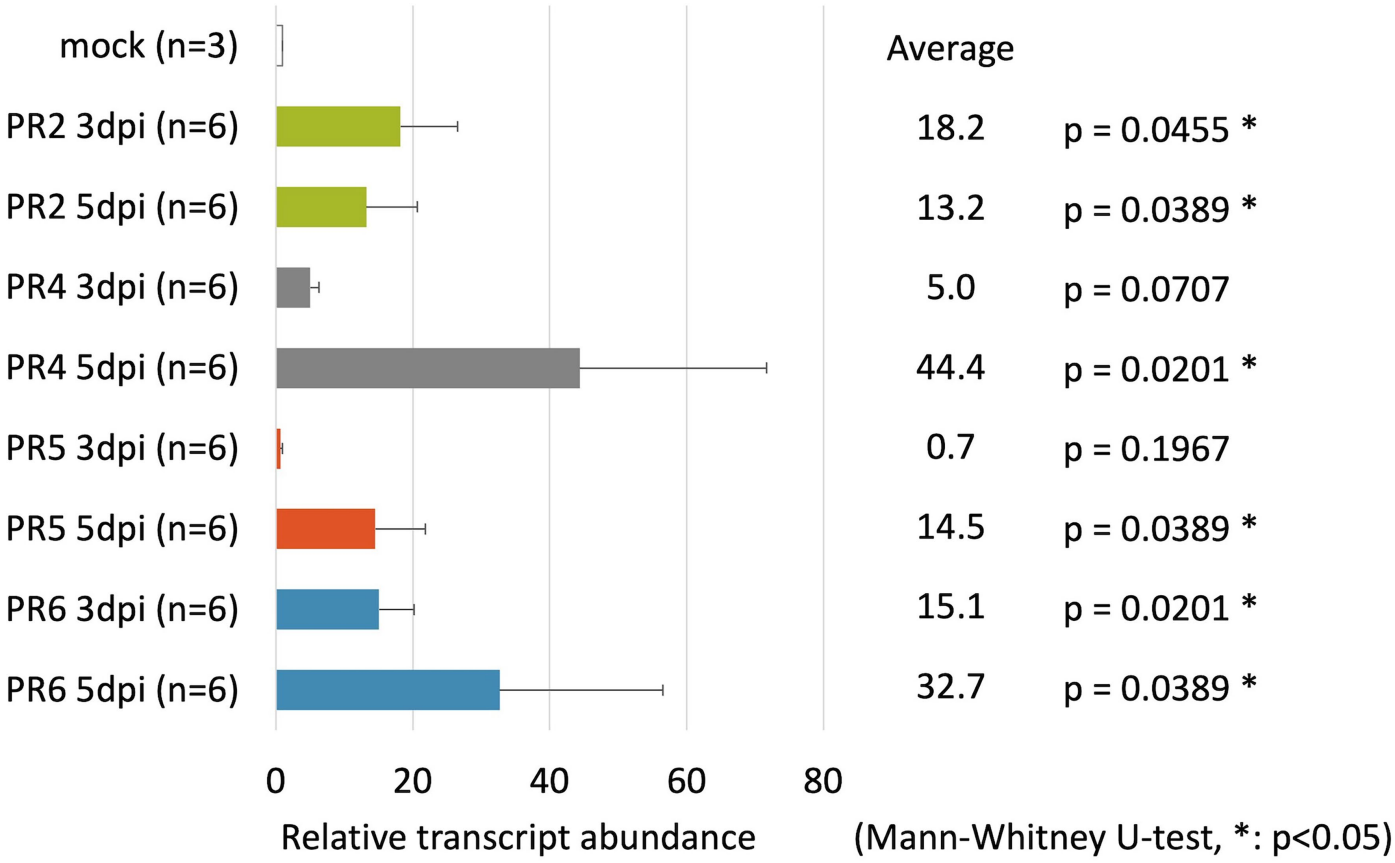
Relative transcript abundance of pathogenesis-related (PR) genes in pine seed embryos following pine wood nematode inoculation. Relative transcript abundance of *PR-2*, *PR-4*, *PR-5*, and *PR-6* genes in pine seed embryos at 3 and 5 days post-infection with *Bursaphelenchus xylophilus*.

To identify suitable marker genes to react to *B. xylophilus* invasion in pine seed embryos, we assayed the expression of six PR genes in pine seed embryos using qRT-PCR. The expression of these PR genes has been suggested to be a good indicator of hypersensitive response that precede pine wilt (Hirao et al., 2012). In this study, the expression levels of four genes (*PR-2*, *PR-4*, *PR-5*, and *PR-6*) were markedly upregulated following inoculation with *B. xylophilus* into pine seed embryos (Figure 2). The expression levels of *PR-2* and *PR-6* in pine seed embryos infected with *B. xylophilus* were > 10-fold higher than those of the mock control (non-inoculated) at 3 and 5 days post-*B. xylophilus* infection (dpi; *p* < 0.05). The expression levels of *PR-4* and *PR-5* were significantly higher than those of the mock control only at 5 dpi (*p* < 0.05). Expression data for *PR-1b* and *PR-3* were omitted from Figure 2 due to their unstable expression in pine seed embryos, irrespective of the presence of *B. xylophilus*. Therefore, we used *PR-2*, *PR-4*, *PR-5*, and *PR-6* as marker genes for subsequent molecular functional analyses.

### Bx-TH1, Bx-TH2, and Bx-GH30 Induced PR Gene Expression in Pine Seed Embryos

**Figure 3:**
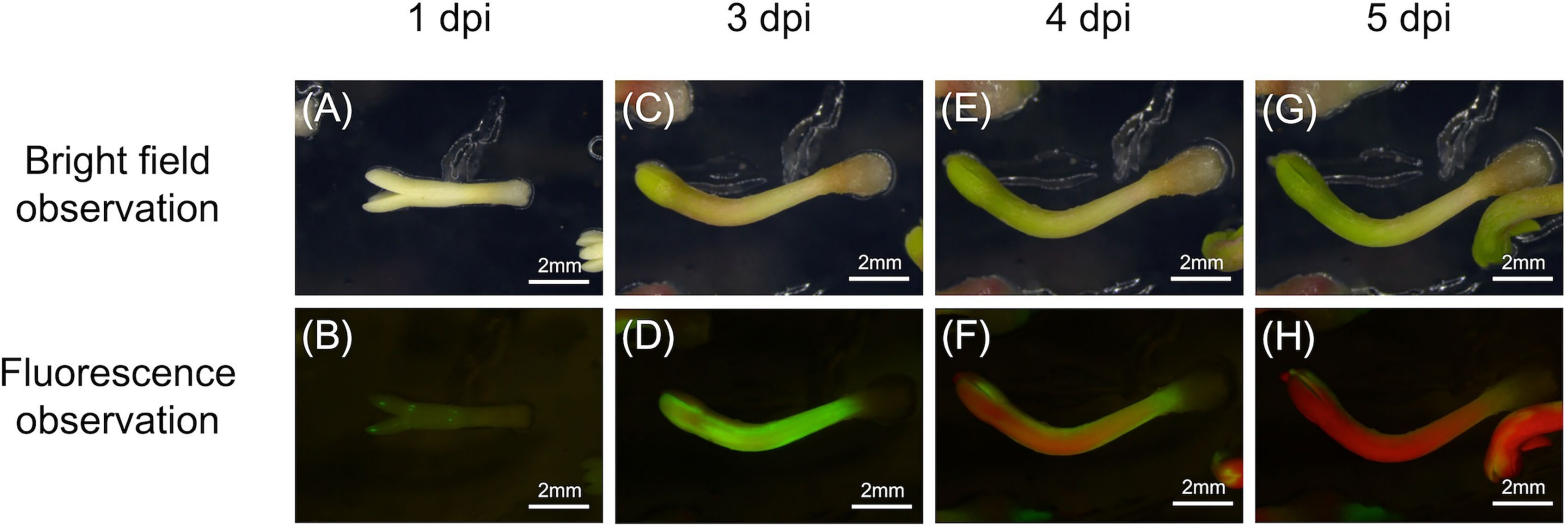
Green fluorescence protein (GFP) detection in pine seed embryos. GFP fluorescence in pine seed embryos at **(A,B)** 1, **(C,D)** 3, **(E,F)** 4, and **(G,H)** 5 days after particle bombardment with *Apple latent spherical virus* (ALSV) vectors. **(A,C,E,G)** Bright-field observations; **(B,D,F,H)** fluorescence observations.

**Figure 4:**
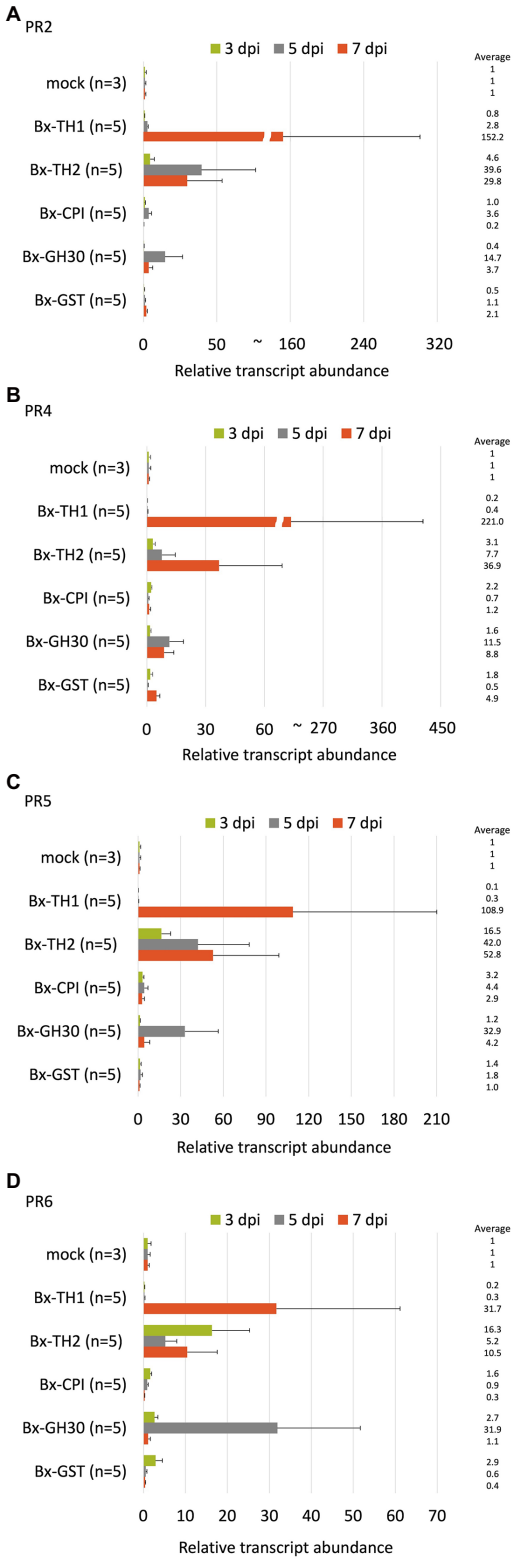
Relative transcript abundance of PR genes between pine seed embryos expressing pathogenic candidate proteins and mock control. Expression levels of **(A)** *PR-2*, **(B)** *PR-4*, **(C)** *PR-5*, and **(D)** *PR-6* in pine seed embryos expressing pathogenic candidate proteins, compared with mock control, using the reference gene elongation factor 1-alpha to normalize results. No statistically significant difference was observed ( *p* > 0.05, Mann-Whitney U-test).

**Figure 5:**
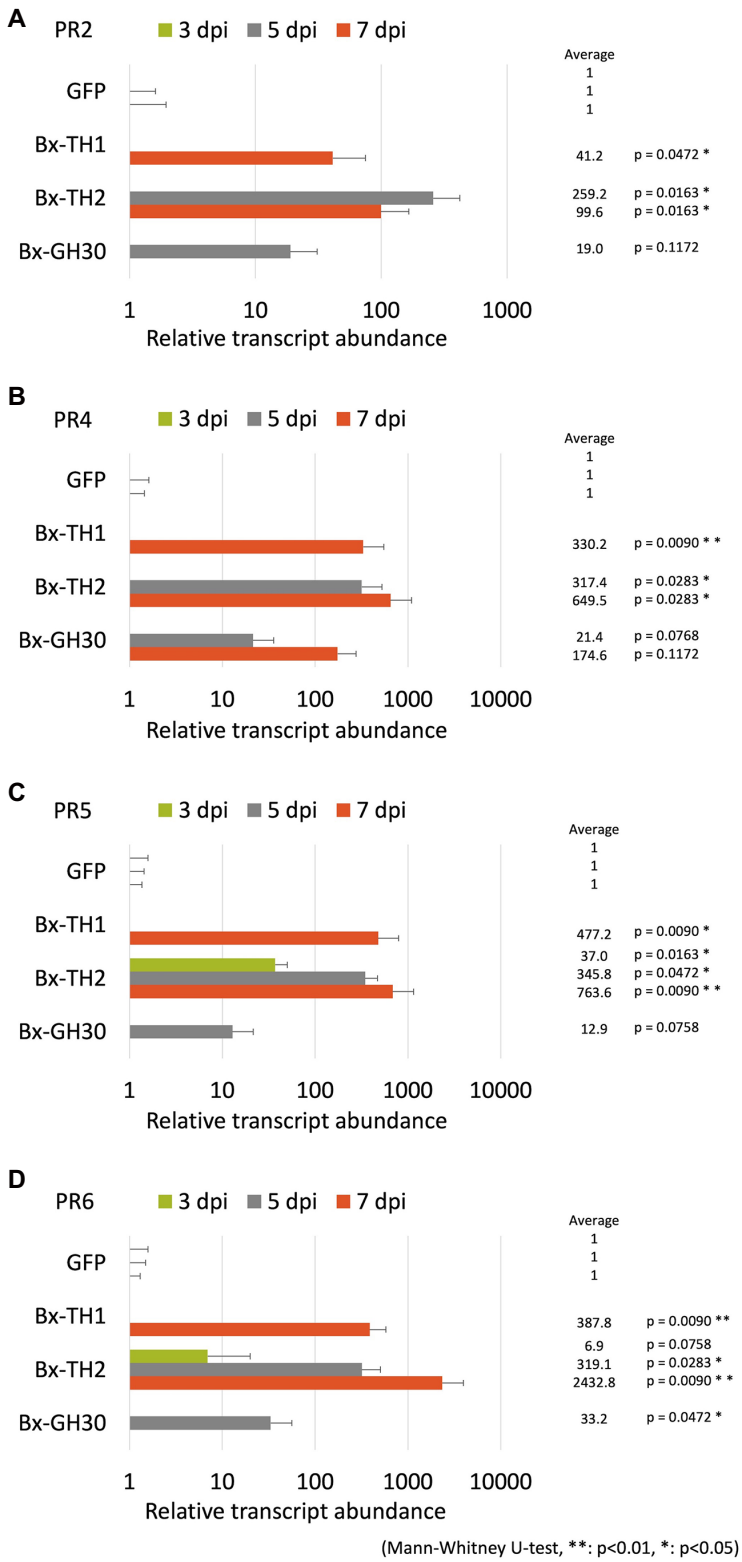
Relative transcript abundance of PR genes between pine seed embryos expressing pathogenic candidate proteins and GFP control. Expression levels of **(A)** *PR-2*, **(B)** *PR-4*, **(C)** *PR-5*, and **(D)** *PR-6* in pine seed embryos expressing pathogenic candidate proteins, compared with GFP control, using the amount of viral RNA detected to normalize results. *p < 0.05 and **p < 0.01 (Mann-Whitney U-test).

To determine whether *B. xylophilus* secreted proteins causing cell death in tobacco could induce the expression of PR genes in pine seed embryos, we constructed and transformed six ALSV vectors, coding sequences of *Bx-TH1*, *Bx-TH2*, *Bx-CPI*, *Bx-GH30*, *Bx-GST*, and *GFP* (control) in pine seed embryos. GFP fluorescence in pine seed embryos was observed from 1 dpi after particle bombardment with ALSV vectors (Figure 3), and almost no GFP signal was observed at 7 dpi. To analyze the function of pathogenic *B. xylophilus* candidate proteins, we investigated the expression of four PR genes (*PR-2*, *PR-4*, *PR-5*, and *PR-6*) at 3, 5, and 7 dpi after ALSV vector particle bombardment using qRT-PCR. Expression levels of the four PR genes in pine seed embryos expressing Bx-TH1, Bx-TH2, and Bx-GH30 were > 5-fold higher than those of mock following normalization by the reference gene elongation factor 1-alpha (Figure 4) and GFP controls following normalization of expression levels according to the amount of viral RNA detected (Figure 5). Though no discrepancies were found between the normalized results using the expression levels of the reference gene elongation factor 1-alpha (mock control) and the amount of viral RNA (GFP control), variation of relative transcript abundance among replicates was lower when normalized with GFP control. When we normalized the gene expression levels by the reference gene elongation factor 1-alpha, no statistically significant difference was observed in relative transcript abundance due to the large variations (Figure 4). When we normalized the gene expression levels by the amount of viral RNA, Bx-TH1 induced significantly higher expression levels of the four PR genes only at 7 dpi than GFP control (*p* < 0.05; Figure 5). Bx-TH2 induced high expression of *PR-5* in pine seed embryos at all time points, and significantly higher expression of *PR-2*, *PR-4*, and *PR-6* at 5 and 7 dpi than GFP control (*p* < 0.05; Figure 5). Bx-GH30 induced high expression of *PR-4* at 5 and 7 dpi, and the other PR genes only at 5 dpi, although these differences were not significant than GFP control, except PR-6 expression at 7 dpi (*p* < 0.05; Figure 5). *Bx-CPI* and *Bx-GST* did not induce PR gene expression in pine seed embryos.

## Discussion

To develop a method for expressing *B. xylophilus* proteins in pine trees, which are its natural hosts, we conducted transient overexpression of five proteins secreted by *B. xylophilus* causing cell death in tobacco in susceptible black pine seed embryos using the ALSV vector and investigated whether a hypersensitive response could be induced by these proteins expression based on the transcript level of PR genes on pine seed embryos.

Previous studies using tobacco adopted cell death as an indicator for screening nematode pathogenic factors; however, exogenous gene expression was found to be transient and insufficient for inducing cell death during *B. xylophilus* protein expression in pine seed embryos using the ALSV vector. Instead, we constructed a GFP expression vector and bombarded ALSV particles into pine seed embryos and clearly observed the GFP signal at 1–7 dpi. This result suggests that pine embryos suppress the expression of foreign genes through post-transcription gene silencing (PTGS). PTGS is commonly observed when plants are transformed using foreign genes (Depicker and Van Montagu, 1997). For example, when ALSV with GFP or yellow fluorescent protein (YFP) was inoculated to tobacco leaves, both types of fluorescence gradually faded after 10 dpi (Takahashi et al., 2007; Yaegashi et al., 2007). Several pine species were transformed inefficiently and unstably using *Agrobacterium*, possibly due to gene silencing, including *P. halepensis* (Tzfira et al., 1996), *P. taeda* (Wenck et al., 1999), *P. strobus* (Levée et al., 1997), and *P. pinaster* (Trontin et al., 2002). Therefore, we focused on PR gene expression in host pine embryos as an indicator for screening nematode pathogenic factors. A previous study showed that the expression of antimicrobial peptides and putative PR genes (e.g., *PR-1b*, *PR-2*, *PR-3*, *PR-4*, *PR-5*, and *PR-6*) was higher in susceptible pine trees than in resistant trees after *B. xylophilus* infection (Hirao et al., 2012). Therefore, we used *PR-1b*, *PR-2*, *PR-3*, *PR-4*, *PR-5*, and *PR-6* as candidate marker genes for *B. xylophilus* invasion into pine seed embryos. We found that the expression levels of PR-2, PR-4, PR-5, and PR-6 were significantly increased in susceptible pine seed embryos following *B. xylophilus* infection (*p* < 0.05). *PR-1b* and *PR-3* expression levels were not stably detected, perhaps because they were too weak for detection by qRT-PCR. *PR-2*, *PR-4*, *PR-5*, and *PR-6* showed similar responses to those in pine seedlings following *B. xylophilus* inoculation; therefore, we used these four genes as indicators of the pine hypersensitive response in subsequent analyses.

Next, we expressed candidate pathogenic *B. xylophilus* molecules in pine seed embryos and assessed their functions by detecting the expression patterns of *PR-2*, *PR-4*, *PR-5*, and *PR-6*. The expression levels of PR genes in pine seed embryos expressing Bx-TH1, Bx-TH2 and Bx-GH30 were > 5-fold higher than those of the mock and GFP controls. This result is consistent with our previous report that these three molecules induced cell death in *N. benthamiana* (Kirino et al., 2020). Further studies with many pathogenic molecules of *B. xylophilus* are necessary to make the consistency of the results obtained in tobacco and pine seed embryo more clear.

The two remaining pathogenic candidate proteins examined in this study (Bx-CPI and Bx-GST) did not increase PR gene expression in pine seed embryos relative to the mock and GFP controls. This result is inconsistent with the previous *N. benthamiana* functional screening result. Woody plants such as pines and herbaceous plants such as *N. benthamiana* differ in structure, lifespan, and defense systems. Generally, the defenses of slow-growing woody plants are typically quantitative and are affected by genetic variation, whereas those of fast-growing herbaceous plants are qualitative and influenced by environmental variation (Massad et al., 2011). This difference in defense systems between pine seed embryos and *N. benthamiana* may have influenced our functional screening results, suggesting that screening using *N. benthamiana* may result in false positives, although results can vary between tobacco and pine seed embryos depending on the type of gene expressed.

Although the assay system established in this study is useful, it has two limitations. First, this method tends to produce large variation in gene expression levels. Expression levels of the four selected PR genes differed among individuals and transgenes at each time point. When qRT-PCR is performed, individual variation in endogenous PR gene expression levels should be considered. Because ALSV vectors were introduced into pine seed embryos biolistically, the amount of vector introduced into each embryo is variable, leading to variation in *B. xylophilus* protein expression. The expression efficiency of each gene introduced into a pine seed embryo may also differ among genes. To reduce this variability in gene expression, we normalized PR gene expression based on the amount of viral RNA in each pine seed embryo (Figure 5), which produced more stable gene expression patterns. The second limitation consists of the differential characteristics between seed embryos and seedlings. Our previous studies showed that changes in the subcellular localization of candidate proteins can alter their ability to induce cell death in tobacco (Kirino et al., 2020; Shinya et al., 2021). As seed embryos are composed of undifferentiated tissue, the localization of *B. xylophilus* protein expressed in seed embryos may differ from that in seedlings. Therefore, to clarify whether candidate molecules that induced hypersensitive responses in seed embryos exhibit the same function in pine seedlings, it is necessary to compare their localization. Furthermore, though many reports have suggested the relationship between the hypersensitive response and pine wilt, there is still no direct evidence to prove it due to technical limitations. The method we have developed using ALSV vectors in this study may contribute to proving this in the future because ALSV vectors have been shown to effectively induce stable virus-induced gene silencing in various plants (e.g., Sasaki et al., 2011).

In summary, we report a novel functional analysis that uses recombinant pathogenic candidate molecules of *B. xylophilus* in host pines. The results of our investigation of tobacco and pine seed embryos suggest that tobacco-based screening for pathogenic *B. xylophilus* molecules may result in false positives. Although the proposed method has limitations that must be resolved in future research, it has potential as a powerful tool for screening pathogenic molecules of *B. xylophilus* in their native host.

## Data Availability Statement

The original contributions presented in the study are included in the article/supplementary material; further inquiries can be directed to the corresponding author.

## Author Contributions

HK, KK, and RS designed the study and wrote the manuscript. HK and KK performed the research and analyzed the data. All authors contributed to the article and approved the submitted version.

## Funding

This study was funded by grants from JSPS Grant-in-Aid for Early-Career Scientists JP19K15853 (to RS), JSPS Grant-in-Aid for JSPS Research Fellow JP21J20926 (to HK), and JST PRESTO grant no. JPMJPR17Q5 (to RS).

## Conflict of Interest

The authors declare that the research was conducted in the absence of any commercial or financial relationships that could be construed as a potential conflict of interest.

## Publisher’s Note

All claims expressed in this article are solely those of the authors and do not necessarily represent those of their affiliated organizations, or those of the publisher, the editors and the reviewers. Any product that may be evaluated in this article, or claim that may be made by its manufacturer, is not guaranteed or endorsed by the publisher.
